# Genetic Abnormalities in the Diagnosis and Treatment of Childhood Acute Myeloid Leukemia: A Prospective Study at Hue Central Hospital, Vietnam

**DOI:** 10.7759/cureus.85864

**Published:** 2025-06-12

**Authors:** Hoa Thi Kim Nguyen, Hao Kiem Tran, Viet Hung Phan, Son Binh Bao Bui

**Affiliations:** 1 Pediatric Department of Oncology, Hematology, and Transplant, Hue Central Hospital, Hue, VNM; 2 Department of Pediatrics, Hue Central Hospital, Hue, VNM; 3 Department of Pediatrics, Hue University of Medicine and Pharmacy, Hue, VNM

**Keywords:** acute myeloid leukemia, children, genetic abnormalities, genetic risk group, treatment outcome

## Abstract

Background: Genetic tests are important in the classification, treatment, and prognosis of acute myeloid leukemia (AML). The present study aimed to detect genetic abnormalities and investigate the correlation between gene abnormalities and the treatment results of childhood AML.

Methods: A descriptive cross-sectional study of 35 children with de novo AML was established between 2017 and 2022 at Hue Central Hospital, Vietnam. Parameters of age, gender, gene fusions, remission, relapse rate, and survival rates were investigated.

Results: The male-to-female ratio was 1.92:1. The mean age was 7.3±4.9 years. The multiplex reverse transcription polymerase chain reaction (RT-PCR) using the HemaVision 28N kit test results showed that 12 (34.3%) patients had genetic abnormalities, of which five (14.2%) patients had AML1/ETO fusion, three (8.6%) had PML/RARA fusion, two (5.7%) had MLL/AF6 fusion, one (2.9%) had KMT2A/MLLT10 fusion, and one (2.9%) had AML1/ETO and BCR/ABL1 fusion. Prognostic grouping according to genetic mutation showed eight (22.9%) patients with a favorable prognosis, 23 (65.7%) patients with an intermediate prognosis, and four (11.4%) patients with a poor prognosis. There were significant relationships between the remission rate and the genetic risk group. The remission rates for poor, intermediate, and good prognosis groups were 25%, 43.5%, and 100%, respectively. However, there were no statistical correlations between the relapse rate, the overall survival rate, and the event-free survival rate with the genetic risk group.

Conclusions: Genetic abnormalities have a role in the classification, prognosis, and treatment of AML patients. However, treatment outcomes in AML are influenced by multiple factors beyond genetics, including infection-related complications, nutritional status, socioeconomic conditions, supportive care infrastructure, and access to intensive chemotherapy and transplant services. Supportive care plays an important role in the treatment outcome of childhood AML.

## Introduction

Acute myeloid leukemia (AML) is a malignant clonal disorder of hematopoietic progenitor cells characterized by uncontrolled proliferation, impaired differentiation, and accumulation of immature myeloid cells in the bone marrow and peripheral blood. It accounts for approximately 15-20% of pediatric leukemias and remains a significant therapeutic challenge due to its aggressive clinical course and high relapse rates [1-3]. Genetic abnormalities play a central role in the pathogenesis, classification, and prognosis of AML. Specific chromosomal translocations such as t(8;21), inv(16), and t(15;17) are associated with favorable prognoses and are categorized as low-risk. Conversely, abnormalities like monosomy 7, FLT3-ITD, and complex karyotypes are often linked with poor outcomes and classified as high-risk [4]. The most recent World Health Organization (WHO) classification (2022) integrates genetic profiles as a central element in AML diagnosis, underscoring their prognostic significance and therapeutic implications [5]. To detect these abnormalities, genetic testing has become an integral component of AML workup. Techniques such as cytogenetics, fluorescence in situ hybridization (FISH), and polymerase chain reaction (PCR) allow for the precise identification of fusion genes and other pathogenic mutations. In Vietnam, genetic tests on AML patients are carried out at a number of large centers, with the test being used to detect four types of common gene fusions using the reverse transcription (RT)-PCR technique. The Pediatric Center of Hue Central Hospital was established in 2013 on the basis of the original pediatric department. Children with blood cancer have been treated in Hue since 2005 [6]. However, until 2018, the Pediatric Center of Hue Central Hospital could do multiplex RT-PCR using the HemaVision 28N kit genetic analysis for AML patients. In addition to molecular characteristics, demographic factors such as age and gender have been investigated in the literature for their potential association with treatment outcomes in AML. However, our study did not evaluate age or gender in relation to the clinical endpoint. Instead, we focused specifically on exploring the correlation between genetic risk classification and treatment outcomes.

This study aims to detect genetic abnormalities and assess the relationship between genetic risk groups, identified using the HemaVision 28N RT-PCR panel and key treatment responses, including complete remission (CR) after induction, relapse, overall survival (OS), and event-free survival (EFS) in pediatric patients diagnosed with AML at Hue Central Hospital. By strengthening the understanding of molecular risk factors in this context, we hope to contribute to improved stratification and management strategies for childhood AML in Vietnam.

## Materials and methods

All patients underwent multiplex RT-PCR using the HemaVision 28N kit. A DNA diagnostic RT-PCR assay is capable of identifying 28 recurrent chromosomal translocations and over 145 clinically relevant fusion transcripts associated with hematologic malignancies. The test employs a nested RT-PCR design to enhance sensitivity and specificity, making it a reliable method for rapid profiling and risk stratification in pediatric AML cases.

The study materials included 35 children diagnosed with AML treated at the Pediatric Center of Hue Central Hospital with the AML protocol (Table 1) between November 2017 and May 2022.

**Table 1:** Treatment protocol for childhood AML at Hue Central Hospital ATRA: all-trans retinoic acid; MTX: methotrexate; AML: acute myeloid leukemia

Drugs	Dose and regimen
Induction phase	
Cytarabine	100 mg/m^2^/day×7 days
Daunorubicin	45 mg/m^2^/day×3 days
BMA at day 14: If the patient does not achieve remission, blast cells > 20%	
Cytarabine	2000 mg/m^2^/12 hours×6 days
BMA at day 14: If the patient does not achieve remission: 5% < blast cells < 20%	
Cytarabine	100 mg/m^2^/day×7 days
Daunorubicin	45 mg/m^2^/day×3 days
Intensification 1	
Cytarabine	1000 mg/m^2^/12 hours×4 days
Daunorubicin	45 mg/m^2^×3 days
Intensification 2	
Cytarabine	1000 mg/m^2^/12 hours×4 days
Etoposide	100 mg/m^2^/day×4 days
Intensification 3: Repeat intensification 1 or	
Cytarabine	3000 mg/m^2^/12 hours×3 days
For AML-M3	
Induction phase	
ATRA	25 mg/m^2^/day
Daunorubicin	45 mg/m^2^/day×3 days
Consolidation (2-3 episodes)	
Daunorubicin	45 mg/m^2^/day×3 days
Cytarabine	1000 mg/m^2^/12 hours×4 days
Maintenance for two years	
ATRA	25 mg/m^2^/day
MTX	15 mg/m^2^/week
Purinethol	75 mg/m^2^/day

Inclusion criteria were as follows: patients diagnosed with AML and treated with the AML protocol (Table 1), those aged <16, and all patients who underwent multiplex RT-PCR using the HemaVision 28N kit genetic analysis and with 28 basic genetic mutations in AML detected.

Criteria for the diagnosis of AML included clinical features, the results of bone marrow morphology, where the leukemic blasts were counted for equal or more than 20% in the marrow space, and immune markers consistent with AML.

Exclusion criteria were as follows: pediatric patients with secondary or relapsed AML and cases where the child and the representative did not agree to participate in the study.

Data were analyzed according to genetic tests, the remission rate, the relapse rate, and the OS and EFS rates. All statistical analysis was performed using IBM SPSS Statistics for Windows, Version 18.0 (Released 2019; IBM Corp., Armonk, New York, United States).

Ethical approval

This study was approved by the Hue Central Hospital Ethics Committee on October 20, 2017, with the approval number 18/NCKH-BVH for both research on AML and acute lymphoblastic leukemia [6]. Consent was obtained from all participants' parents or guardians in this study.

## Results

Among 35 new AML patients, there were 23 males and 12 females; the male-to-female ratio was 1.92:1. The mean age was 7.3±4.9 years. There was no age peak in disease incidence, which increased slightly after the first year of age. Regarding the classification of AML subtypes, M2 and M5 accounted for the highest percentages, 10 (28.6%) for each subtype. The percentages of M6, M3, M1, M0, and M7 were 5 (14.3%), 4 (11.4%), 3 (8.6%), 2 (5.7%), and 1 (2.8%), respectively. 

The results of multiplex RT-PCR using the HemaVision 28N kit showed that 12 (34.3%) patients had genetic abnormalities, of which five (14.2%) had AML1/ETO fusion, three (8.6%) had PML/RARA fusion, two (5.7%) had MLL/AF6 fusion, one (2.9%) had KMT2A/MLLT10 fusion, and one (2.9%) had AML1/ETO and BCR/ABL1 fusion (Table 2).

**Table 2:** Genetic variants in AML AML: acute myeloid leukemia

Gene fusions	Number (%)
AML1/ETO–t(8;21)(q22;q22)	5 (14.2)
AML1/ETO+BCR/ABL1	1 (2.9)
PML/RARA–t(15;17)(q22; q22)	3 (8.6)
MLL/AF6–t(6;11)(q27;q23)	2 (5.7)
KMT2A/MLLT10–t(10;11)(p12;q23)	1 (2.9)
Unexpressed	23 (65.7)
Total	35 (100)

The prognostic grouping according to genetic mutation was as follows: good prognosis 8 (22.9%), intermediate prognosis 23 (65.7%), and poor prognosis 4 (11.4%) (Table 3). Fusion genes play a role in the classification of AML subtypes. Patients with PML/RARA gene fusion were classified as an M3 subtype. Patients with the AML1/ETO gene fusion were mainly in the M2 subtype, and patients with KMT2A/MLLT10 gene fusion were in the M5 subtype.

**Table 3:** Classification of AML groups according to gene fusion AML: acute myeloid leukemia

Classified group according to gene fusions	Number (%)
Poor prognosis (MLL/AF6, KMT2A/MLLT10, BCR/ABL1+AML1/ETO)	4 (11.4)
Intermediate prognosis	23 (65.7)
Good prognosis (AML1/ETO, PML/RARA)	8 (22.9)
Total	35 (100)

In the evaluation after the induction phase, the results showed that 19 (54.3%) patients achieved remission, four (11.4%) patients had partial remission, and three (8.6%) patients did not. Moreover, nine (25.7%) patients died during the induction phase (Table 4). The remission rates of people with poor, intermediate, and good prognosis groups were 25%, 43.5%, and 100%, respectively. There was a statistically significant correlation between genetic abnormalities and the remission rate (p=0.03) (Table 5). We used Fisher's exact test because of the small samples.

**Table 4:** The status of remission after the induction phase

The status of remission	Number (%)
Remission	19 (54.3)
Partial remission	4 (11.4)
No remission	3 (8.6)
Death	9 (25.7)
Total	35 (100)

**Table 5:** The AML remission rate according to genetic risk group AML: acute myeloid leukemia Fisher's exact test was used due to the small samples

The status of remission	Poor prognosis group	Intermediate prognosis group	Good prognosis group	P-value
	N (%)	N (%)	N (%)	
Remission	1 (25)	10 (43.5)	8 (100)	0.03^*^
Partial remission	2 (50)	2 (8.7)	-	
No remission	-	3 (13)	-	
Death	1 (25)	8 (34.8)	-	-
Total	4 (100)	23 (100)	8 (100)	-

When evaluating the AML relapse rate according to genetic risk groups, the results showed no correlation between them (p=0.8). The relapse rates for patients with poor, intermediate, and good prognosis groups were 66.7%, 41.7%, and 28.6%, respectively (Table 6).

**Table 6:** AML relapse rate based on genetic risk group AML: acute myeloid leukemia

Risk group (relapse status)	Poor prognosis	Intermediate prognosis	Good prognosis	P-value
	N (%)	N (%)	N (%)	
No relapse	1 (33.3)	7 (58.3)	5 (71.4)	0.8
Relapse	2 (66.7)	5 (41.7)	2 (28.6)	
Total	3 (100)	12 (100)	7 (100)	

Regarding the evaluation of the survival rate according to the genetic risk group, the result showed that at the end of the study, out of a total of 35 AML patients, there were 17 patients who had died. The number of surviving patients was 18 (51.4%).

**Figure 1:**
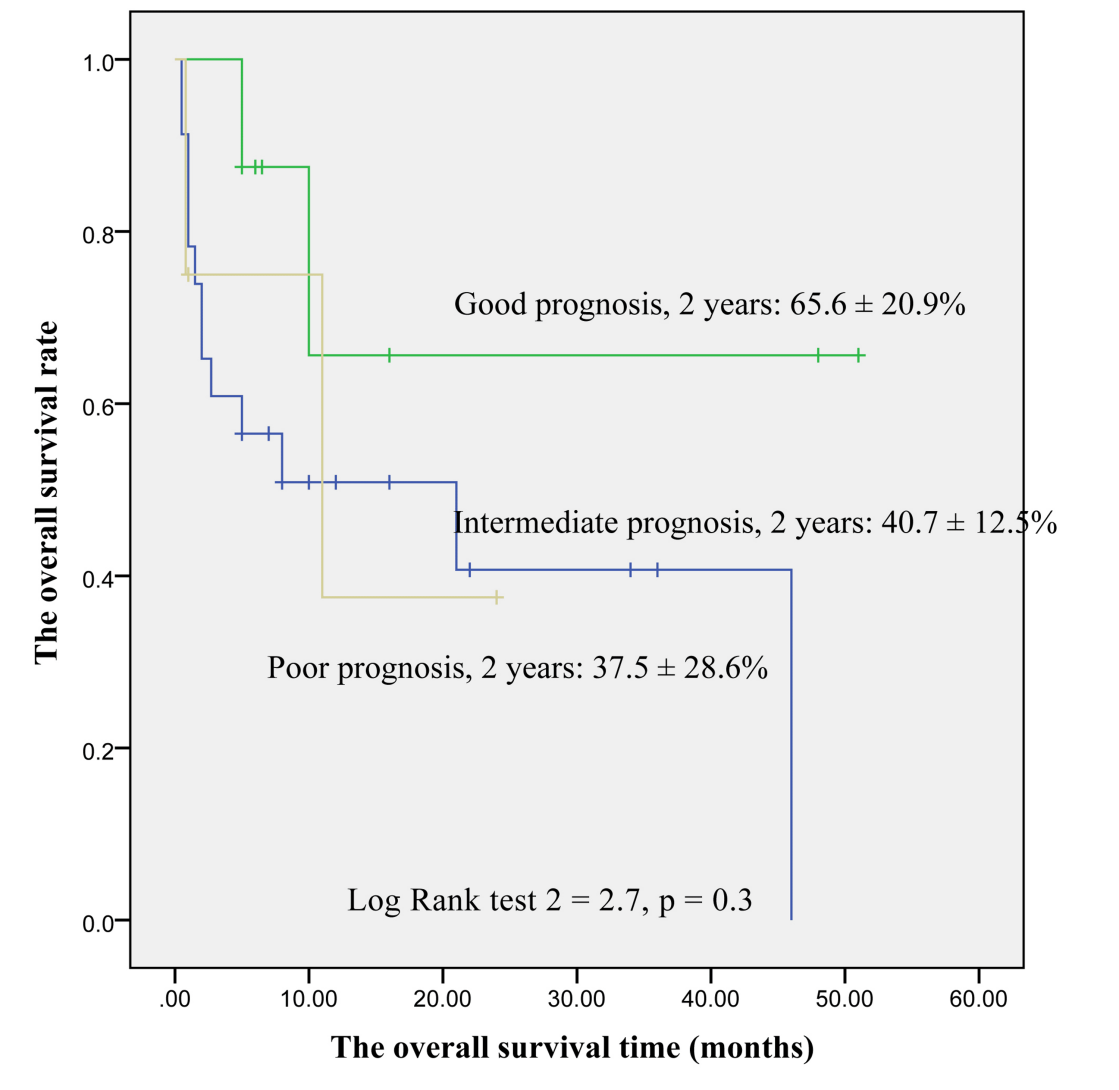
The overall survival rate for AML patients AML: acute myeloid leukemia

**Figure 2:**
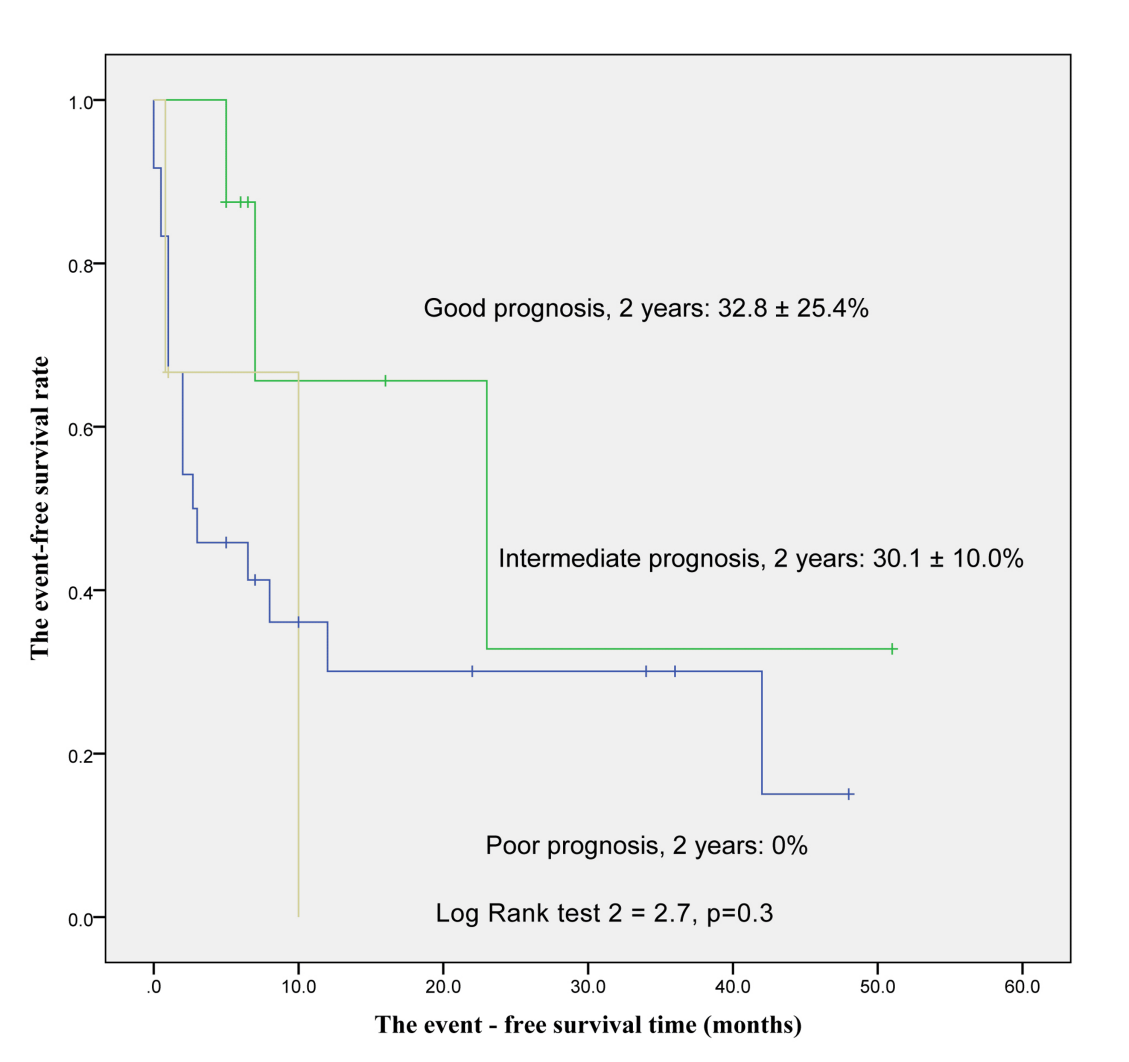
The event-free survival rate for AML patients AML: acute myeloid leukemia

At two years, the poor prognosis group had an OS rate of 37.5±28.6%, and the figures for the intermediate and favorable prognosis groups were 40.7±12.5% and 65.6±20.9%, respectively. However, the difference was not statistically significant (p=0.3) (Figure 1). In the second year, the EFS rates of people experiencing poor, intermediate, and good prognostic groups were 0%, 30.1±10.0%, and 32.8±25.4%, respectively. This difference was not statistically significant (p=0.3) (Figure 2).

## Discussion

The observed male predominance (M:F=1.92:1) in our cohort is consistent with previous studies [5,6]. However, gender is not currently considered an independent risk factor for AML incidence or outcome, and this difference may result from population-based variation or chance [7,8]. The mean age was 7.3±4.9 years, similar to some studies by Juliusson et al. and Deschler and Lübbert [9,10]. Regarding the classification of AML subtypes, M2 and M5 accounted for the highest percentages (28.6% for each subtype), which was similar to the results of Pui's and Shahab and Raziq's studies [7,11].

The results of the multiplex RT-PCR test using the HemaVision 28N kit showed that there were 34.3% of patients with genetic abnormalities, including 14.2% of patients with AML1/ETO, 8.6% of patients with PML/RARA, 5.7% of patients with MLL/AF6, 2.9% of patients with KMT2A/MLLT10, and 2.9% of patients with AML1/ETO and BCR/ABL1 fusion. A new point in our result was that one patient had two fusion genes: AML1/ETO and BCR/ABL1. This was a special case and was reported for the first time in Vietnam. For AML patients, the presence of the BCR/ABL1 fusion gene is rare [12]. The combination of AML1/ETO and BCR/ABL1 is extremely rare. According to foreign literature, there are a few cases having AML1/ETO and BCR/ABL1. And these patients will have a poor prognosis [13]. Besides that, our result also found two fusion genes (MLL/AF6 and KMT2A/MLLT10) which have not been reported in Vietnamese children yet. According to Quessada et al.'s research, the percentages of two gene fusions, MLL/AF6 and KMT2A/MLLT10, were 1-2% and 2-3%, respectively [8]. These mutations are associated with MLL rearrangement and have a poor prognosis. Therefore, detecting gene fusions in AML is critically important for accurate risk stratification, prognosis, and guiding therapeutic decisions.

Based on genetic abnormalities, Quessada et al., Rubnitz and Inaba, and Pui et al. classified patients into three risk genetic groups: favorable, intermediate, and poor prognosis [8,14,15]. In our result, there were 22.9% of patients with a good prognosis, 65.7% of patients with an intermediate prognosis, and 11.4% of patients with a poor prognosis.

The rate of remission after the induction phase for AML was 54.3%; 11.4% of patients had partial remission, and 8.6% of patients did not achieve remission. There, 25.7% of patients passed away during the induction phase. The rate of remission in our study was lower than that of Waack et al.'s study and Bui et al.'s study rates of 84.7% and 89.4%, respectively [16,17]. This issue reflected that supportive care plays an important role in AML treatment. The infection rate was still high in my hospital, and it caused failure in treatment for childhood AML. And we need to improve. The successful treatment for AML patients is still limited; therefore, numerous new therapeutic strategies have been developed globally to improve treatment outcomes for pediatric AML patients [18]. Regarding the correlation between genetic abnormalities and the rate of remission in childhood AML, there was a statistically significant correlation between them (p=0.03). The rates of remission in poor, intermediate, and good prognosis groups were 25%, 43.5%, and 100%, respectively. The results of the present study regarding the remission rate were consistent with findings from previous studies conducted by Pui, Rubnitz and Inaba, and Singh et al., which also reported higher remission rates in patients with AML1/ETO and PML/RARA fusion genes [13-15]. These studies showed that AML1/ETO and PML/RARA were associated with favorable prognoses and PML/RARA had a good response with all-trans retinoic acid (ATRA) drug, while MLL/AF6 mutation was associated with a poor prognosis and a lower remission rate. The combined existence of BCR/ABL1 and AML1/ETO gene fusion had a poor prognosis, even if the patient carried AML1/ETO gene fusion, which was associated with a good prognosis [13-15].

The relapse rates of the poor, intermediate, and good prognosis groups were 66.7%, 41.7%, and 28.6%, respectively. The difference did not have statistical significance (p=0.8). In AML treatment, multiple factors can influence the relapse rate aside from gene fusions. These include high infection rates during intensive chemotherapy, delayed treatment intervals due to supportive care limitations [17], the patient's pre-transplant condition, minimal residual disease (MRD) status, cytogenetic complexity, and AML subtype such as M4/M5 [19,20]. With the use of intensive chemotherapy, the infection rate was high. Prolonged infections will delay treatment time for patients, affecting treatment results and relapse rates. Therefore, supportive care in AML plays an important role. According to Harris et al., there are some factors that predict relapse in AML after transplant: the condition of the bone marrow and the patient before transplant, genetic abnormalities, and subtype M4/M5 [19].

The OS rates after two years for childhood AML with poor, intermediate, and favorable prognosis risk groups were 37.5±28.6%, 40.7±12.5%, and 65.6±20.9%, respectively. The difference was not statistically significant (p=0.3). The EFS rates for people experiencing poverty, intermediate, and favorable prognosis risk groups after two years were 0%, 30.1±10.0%, and 32.8±25.4%. However, there is no statistical difference (p=0.2).

Our results differed from the studies by Cho et al., Pui et al., and Rubnitz and Inaba, who illustrated that there was a correlation between the genetic risk group and overall EFS [14,15,20]. AML treatment is so complicated, and supportive care plays an important role in controlling infection and reducing mortality is very necessary. In our hospital, the present study did not have isolated rooms for childhood AML patients, and supportive care had some limitations. So, the result had some differences. The present study should improve the quality of AML treatment as soon as possible.

In our research, the present study had some limitations because it did a genetic test but did not perform chromosome analysis, so the classification could be missed. In the future, the present study should combine genetic tests and chromosome analysis tests to classify genetic risk groups for childhood AML patients.

## Conclusions

This study highlights the critical role of genetic abnormalities in the classification, prognosis, and initial treatment response in childhood AML. A statistically significant correlation was observed between genetic risk groups and remission rates, reinforcing the value of molecular diagnostics in risk stratification and treatment planning. However, no significant associations were found between genetic risk and relapse rate, OS, or EFS. These findings underscore the multifactorial nature of AML treatment outcomes, especially in resource-limited settings. Inadequate supportive care, particularly in managing infections, may contribute significantly to treatment failure and mortality. Therefore, in addition to advancing genetic diagnostics, enhancing supportive care infrastructure remains essential to improving long-term outcomes for children with AML in Vietnam and similar settings.

## References

[1] Freireich EJ, Wiernik PH, Steensma DP (2014). The leukemias: a half-century of discovery. J Clin Oncol.

[2] Inaba H, Greaves M, Mullighan CG (2013). Acute lymphoblastic leukaemia. Lancet.

[3] Zwaan CM, Kolb EA, Reinhardt D (2015). Collaborative efforts driving progress in pediatric acute myeloid leukemia. J Clin Oncol.

[4] Meena JP, Makkar H, Gupta AK (2022). A comprehensive analysis of cytogenetics, molecular profile, and survival among pediatric acute myeloid leukemia: a prospective study from a tertiary referral center. Am J Blood Res.

[5] Khoury JD, Solary E, Abla O (2022). The 5th edition of the World Health Organization classification of haematolymphoid tumours: myeloid and histiocytic/dendritic neoplasms. Leukemia.

[6] Tran HK, Nguyen HTK, Phan VH (2024). Research of genetic abnormalities in diagnosis and treatment of childhood acute lymphoblastic leukemia at Hue Central Hospital. Ann Clin Anal Med.

[7] Shahab F, Raziq F (2014). Clinical presentations of acute leukemia. J Coll Physicians Surg Pak.

[8] Quessada J, Cuccuini W, Saultier P, Loosveld M, Harrison CJ, Lafage-Pochitaloff M (2021). Cytogenetics of pediatric acute myeloid leukemia: a review of the current knowledge. Genes (Basel).

[9] Juliusson G, Antunovic P, Derolf A (2009). Age and acute myeloid leukemia: real world data on decision to treat and outcomes from the Swedish Acute Leukemia Registry. Blood.

[10] Deschler B, Lübbert M (2006). Acute myeloid leukemia: epidemiology and etiology. Cancer.

[11] Pui CH (1995). Childhood leukemias. N Engl J Med.

[12] Neuendorff NR, Schwarz M, Hemmati P (2015). BCR-ABL1+ acute myeloid leukemia: clonal selection of a BCR-ABL1- subclone as a cause of refractory disease with nilotinib treatment. Acta Haematol.

[13] Singh MK, Gupta R, Rahman K, Kumar S, Sharma A, Nityanand S (2017). Co-existence of AML1-ETO and BCR-ABL1 transcripts in a relapsed patient of acute myeloid leukemia with favorable risk group: a coincidence or clonal evolution?. Hematol Oncol Stem Cell Ther.

[14] Rubnitz JE, Inaba H (2012). Childhood acute myeloid leukaemia. Br J Haematol.

[15] Pui CH, Carroll WL, Meshinchi S, Arceci RJ (2011). Biology, risk stratification, and therapy of pediatric acute leukemias: an update. J Clin Oncol.

[16] Waack K, Schneider M, Walter C (2020). Improved outcome in pediatric AML - the AML-BFM 2012 study. Blood.

[17] Bui HT, Nguyen QH, Nguyen VD (2018). Clinical and treatment outcome of childhood acute myeloid leukemia at Children Hospital's Number 2. Ho Chi Minh Med J.

[18] Reinhardt D, Antoniou E, Waack K (2022). Pediatric acute myeloid leukemia-past, present, and future. J Clin Med.

[19] Harris AC, Kitko CL, Couriel DR (2013). Extramedullary relapse of acute myeloid leukemia following allogeneic hematopoietic stem cell transplantation: incidence, risk factors and outcomes. Haematologica.

[20] Cho EK, Bang SM, Ahn JY (2003). Prognostic value of AML1/ETO fusion transcripts in patients with acute myelogenous leukemia. Korean J Intern Med.

